# Molecular mechanisms of inflammation and tissue injury after major trauma-is complement the "bad guy"?

**DOI:** 10.1186/1423-0127-18-90

**Published:** 2011-11-30

**Authors:** Miriam D Neher, Sebastian Weckbach, Michael A Flierl, Markus S Huber-Lang, Philip F Stahel

**Affiliations:** 1 Department of Orthopaedic Surgery, University of Colorado Denver, School of Medicine, Denver Health Medical Center, 777 Bannock Street, Denver, CO 80204, USA; 2 Department of Orthopaedic Trauma, Hand, Plastic, and Reconstructive Surgery, University Hospital Ulm, Steinhövelstraße 9, D-89075 Ulm, Germany; 3 Department of Neurosurgery, University of Colorado Denver, School of Medicine, Denver Health Medical Center, 777 Bannock Street, Denver, CO 80204, USA

## Abstract

Trauma represents the leading cause of death among young people in industrialized countries. Recent clinical and experimental studies have brought increasing evidence for activation of the innate immune system in contributing to the pathogenesis of trauma-induced sequelae and adverse outcome. As the "first line of defense", the complement system represents a potent effector arm of innate immunity, and has been implicated in mediating the early posttraumatic inflammatory response. Despite its generic beneficial functions, including pathogen elimination and immediate response to danger signals, complement activation may exert detrimental effects after trauma, in terms of mounting an "innocent bystander" attack on host tissue. Posttraumatic ischemia/reperfusion injuries represent the classic entity of complement-mediated tissue damage, adding to the "antigenic load" by exacerbation of local and systemic inflammation and release of toxic mediators. These pathophysiological sequelae have been shown to sustain the systemic inflammatory response syndrome after major trauma, and can ultimately contribute to remote organ injury and death. Numerous experimental models have been designed in recent years with the aim of mimicking the inflammatory reaction after trauma and to allow the testing of new pharmacological approaches, including the emergent concept of site-targeted complement inhibition. The present review provides an overview on the current understanding of the cellular and molecular mechanisms of complement activation after major trauma, with an emphasis of emerging therapeutic concepts which may provide the rationale for a "bench-to-bedside" approach in the design of future pharmacological strategies.

## Introduction

Despite significant advances in injury prevention, prehospital resuscitation strategies, and modern intensive care, trauma remains the main cause of death in young people in the United States, resulting in more years of potential life lost before the age of 75 years than any other disease [1-4]. Until present, the pathophysiology of major trauma remains poorly understood [5,6]. In principle, the pathophysiological sequelae of major injuries are characterized by the initial traumatic impact (so-called "first hit"), followed by a cascade of subsequent immunological reactions, which render the patient susceptible to a potentially detrimental "second hit" insult [7]. The activation of innate immune response mechanisms has been characterized as a crucial event initiating the early phase of hyperinflammation within hours to days after major trauma [6-8]. While innate immunity is classically considered to be the immediate "first line of defense" against non-self antigens (e.g. infectious pathogens), a traumatic insult can induce a similarly potent acute inflammatory response [9-13]. The trauma-induced immune response may be limited locally, as in isolated injuries, or result in a massive systemic immune activation, as in patients with multiple injuries [1]. The endogenous triggers of trauma-associated inflammation have been thoroughly investigated and characterized in recent years [7,14]. The so-called "first hit" induced by a traumatic impact leads to the appearance of an arsenal of "damage-associated molecular patterns" (DAMPs) that are recognized by receptors of immune cells [15]. DAMPs represent a recently characterized large superfamily of danger signals which can activate innate immune responses after trauma or trauma-induced complications, such as infection and sepsis [7,16]. The DAMP family of danger signals includes the so-called "pathogen-associated molecular patterns" (PAMPs) and molecules termed "alarmins" [17]. The list of molecules belonging to the DAMP family has been increasing dramatically in recent years, and their pathophysiological function in mediating trauma-induced inflammation is far from being fully understood [18]. PAMPs represent a heterogenic entity of recently described inflammatory molecules related to the innate immune system [17,19]. These microbial molecules are recognized by the immune system as foreign due to their characteristic molecular patterns. In contrast, the so-called "alarmins" represent the correlate of PAMPs for all non-pathogen-derived danger signals which originate from tissue injury [17]. This heterogeneic group of danger molecules is capable of activating innate immune responses in response to tissue damage and cell injury. The alarmins comprise the "heat-shock proteins" (HSPs), annexins, defensins, as well as "classical" markers of tissue injury, such as the S100 protein and the high mobility group box 1 (HMGB1) protein [17,20]. Immunologically competent cells recognize both PAMPs and DAMPs through multiligand receptors expressed on their surfaces, such as Toll-like receptors (TLRs) [21,22].

**Figure 1:**
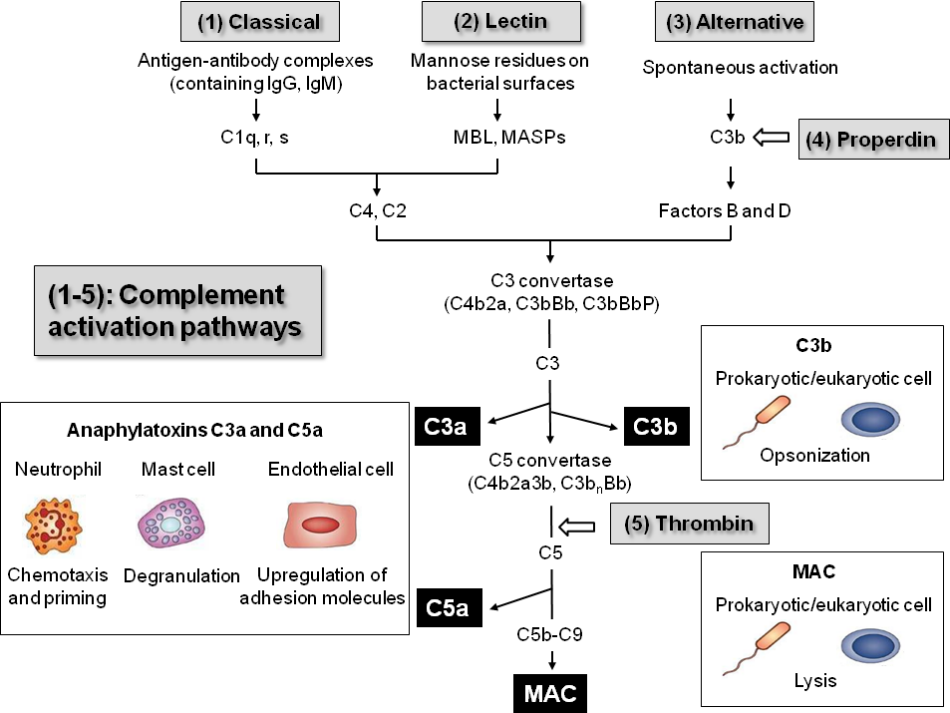
**Overview on the complement activation pathways and biological effects mediated by complement products**. See text for details and explanations.

The very early stage after tissue trauma is characterized by activation of cellular and molecular effectors of the innate immune system, including complement activation and recruitment and activation of neutrophils (polymorphonuclear leukocytes; PMNL) [6,7]. The complement system appears to represent the crucial effector of innate immune responses in the early phase after major trauma [23-25]. Once the cascade is activated through one of three (five) established pathways (Figure 1), complement plays a critical role in the elimination of invading pathogens by opsonization for phagocytosis (C3b, C4b), chemotaxis of leukocytes (C3a, C5a), and by direct lysis of pathogens through the membrane attack complex (MAC, C5b-9) [23,26,27]. The generation of anaphylatoxins C3a and C5a provides potent chemoattractants for phagocytes and neutrophils, and recruit these immune cells to the site of injury [24,28,29]. The anaphylatoxins further induce degranulation of mast cells, basophils and eosinophils and mediate the hepatic acute-phase response [30,31]. Finally, the generation of C5b by cleavage of C5 initiates the terminal complement pathway with MAC formation. The MAC forms through the self-association of C5b along with C6 through C9 and leads to the formation of a large membranolytic complex capable of lysing prokaryotic and eukaryotic cells [32]. Multiple previous studies have unequivocally shown that trauma activates complement, both locally at the site of injury, and systemically. Early studies in the 1980s revealed that the complement cascade is activated at the level of C3 in serum of trauma patients, and the extent of activation correlates with the severity of injury [33,34].

The neutrophil (or PMNL) has been established as the cellular counterpart to the humoral immune response mediated by complement activation, and represents a "key effector" cell of the early posttraumatic immune response. Within minutes, and up to several days after injury, neutrophils play an important role in mounting the immunological defense and the debridement of injured tissue. Primed neutrophils are capable of mediating an inflammatory response, characterized by release of cytokines, chemokines, reactive oxygen species, and tissue-toxic enzymes, such as myeloperoxidase and elastase [20,35]. Aside from the beneficial role of neutrophils in host-defense and clearance of damaged tissue after trauma, excessive priming and cellular PMNL activation may lead to an overwhelming inflammatory response and "innocent bystander" injury to host tissue [35,36]. Uninjured tissue may become damaged by the local release of toxic metabolites and enzymes, thus contributing to remote organ injury (e.g. to brain and lungs), by contributing to tissue edema and secondary tissue damage [12,35,37-39].

Based on the delicate balance between protection and harm, the posttraumatic inflammatory response has been rightfully termed a "double-edged sword" [40-42]. The present review will outline the current understanding of complement activation and regulation after major trauma, with a focus on specific injury patterns, including musculoskeletal trauma, ischemia/reperfusion, chest and brain injuries. We will furthermore discuss potential new pharmacological strategies related to the targeted inhibition of complement, which may shed some hope into the design of new immunomodulatory treatment modalities for severely injured patients in the future.

### Complement activation and effector functions

The complement system represents one of the phylogenetically oldest cascade systems of the body, consisting of a proteolytic cascade of more than 30 soluble and surface-bound proteins that can be activated by the classical, the lectin and the alternative pathway [32,43,44]. Recently, two additional complement activation pathways have been described, i.e. the properdin and the thrombin pathways, both of which will be discussed in more detail below. Figure 1 depicts a rough schematic of the so far known complement activation pathways and of the biological functions of activated complement components. In brief, the three main activation pathways converge in the formation of enzymatic complexes termed the C3 convertases and C5 convertases, which cleave the two main components of the complement system, C3 and C5. The two proteolytic fragments generated by the action of the convertases are the anaphylatoxins C3a and C5a. Both can trigger proinflammatory signaling through binding to their corresponding receptors, the C3a receptor (C3aR) and C5a receptor (C5aR and C5L2), on various myeloid and non-myeloid cells [28,29,45,46]. C5a is a powerful chemoattractant for neutrophils that recruits immune cells to the site of injury and activates cellular attack mechanisms like oxidative burst and lysosomal enzyme release [47,48]. Furthermore, the anaphylatoxins contribute to the degranulation of mast cells and basophils, induce the expression of adhesion molecules on endothelial cells, cause smooth-muscle contraction and enhance the acute phase response of the liver [48]. The cleavage of C3 by C3 convertases leads to the generation of a second major fragment, C3b, which acts as an opsonin facilitating the removal of bacteria and cell detritus by phagocytic cells [49]. Finally, the formation of C5b by cleavage of C5 initiates the assembly of a multimolecular complex, the MAC (C5b-9), that perforates membranes of bacteria and nucleated cells and causes rapid cell lysis and death [45,50,51].

Recently, a second initiation mechanism of the alternative activation pathway was described, termed the properdin pathway [52]. Properdin is capable of recognizing several DAMPs and PAMPs on foreign and apoptotic cells, thus allowing C3 convertase assembly on the target surface [32,52]. Properdin also functions as a stabilizer for C3 convertase complexes of the alternative pathway. In addition to properdin, a fifth complement activation pathway has been described, which identified the clotting factor thrombin as a C5 convertase. This notion was supported by the observation that thrombin is capable of generating C5a in the absence of C3, thus providing a direct link between the complement and coagulation system [53,54].

### Traumatic brain injury

Traumatic brain injury (TBI) induces a profound inflammatory response that contributes to brain edema, neuronal cell death, and adverse outcome [55-57]. Posttraumatic activation of the complement cascade has been shown to play a pivotal role in the development of secondary brain injury (Table 1) [10,12,23,24,58,59]. Multiple experimental and clinical studies have revealed elevated levels of complement components and complement activation fragments in serum, cerebrospinal fluid (CSF), and brain parenchyma after head injury [12,23,60,61]. Intracerebral complement deposition after TBI derives either from an altered permeability of a dysfunctional blood-brain barrier (BBB), or from posttraumatic biosynthesis of complement components by resident and infiltrating cells of the central nervous system (CNS) [12,62-64]. Most studies have focused on the central complement component C3, and on the potential neuroprotective effects of inhibiting C3 convertases, the level at which the three main activation pathways merge, thus inhibiting downstream complement activation. Clinical studies revealed elevated C3 levels in the CSF of patients with severe TBI [65]. Experimental brain injury models described intracerebral PMNL infiltration and concomitant accumulation of complement C3 in cortical and hippocampal brain sections after experimental TBI in rats [66]. In those studies, C3 accumulation was significantly related to places of intracerebral cell death and to increased intracerebral myeloperoxidase activity [66]. In accordance with these findings, C3-deficient mice were found to have lower neutrophil extravasation and cerebral lesion volumes in a freeze model of brain injury [67]. In light of the central role of C3 and downstream complement activation fragments in the pathophysiology of TBI, much emphasis has been recently devoted to elucidating therapeutic aspects of C3 convertase inhibition, in various experimental model systems [68-72]. Genetically engineered mice, either deficient in the C3 gene, or with transgenic CNS-restricted overexpression of *Crry *- a soluble inhibitor of C3 convertases in mice-showed a significant extent of neuroprotection after brain injury, compared to wild-type animals [67,70]. The *GFAP-sCrry *transgenic mice showed a significantly improved neurological outcome and an attenuated extent of posttraumatic BBB dysfunction in a model of closed head injury [70]. Based on these insights, the concept of *Crry-*mediated neuroprotection was extrapolated to a pharmacological approach, by posttraumatic injection of a recombinant chimeric *Crry-Ig *molecule in the same model of closed head injury [71]. The systemic injection of *Crry-Ig *during an early therapeutic "window of opportunity" within one hour to 24 hours after trauma resulted in a significant neurological improvement and reduced extent of neuronal cell death, compared to vehicle-injected control mice [71]. A similar therapeutic approach was tested in a fluid percussion model of brain injury, using recombinant Vaccinia virus complement control protein (VCP), a potent inhibitor of alternative and classical pathway C3 convertases [69,72]. In these studies, the intracranial administration of VCP mediated neuroprotective effects related to posttraumatic preservation of spatial memory, as compared to vehicle-injected controls [69,72].

**Table 1:** Insights from experimental complement inhibition based on genetically engineered mice and pharmacological approaches in models of traumatic brain injury (TBI).

Complement inhibitor/mouse strain	Inhibited complement molecule	Affected complement pathway	Inhibition-induced effects	Reference
***C3^-/- ^*mice**	C3	Classical, alternative, lectin	Reduction of neutrophil extravasation, injury sizes and chemokine expression.	Sewell et al., 2004 [67]

***C4^-/- ^*mice**	C4	Classical, lectin	Decrease of motor deficits and brain lesion size.	You et al., 2007 [81]

***Factor B^-/- ^*mice, anti-factor B monoclonal Ab**	Factor B	Alternative	Attenuation of cerebral tissue damage and neuronal apoptosis, upregulation of anti-apoptotic mediators, down-regulation of pro-apoptotic markers.	Leinhase et al., 2006, 2007 [79,80]

***CD59a^-/- ^*mice**	CD59a	Terminal	Exacerbated tissue injury in CD59a-deficient mice, implying MAC-mediated secondary neuronal cell death.	Stahel et al., 2009 [88]

**C1-INH**	C1r/s, MASPs, C3b	Classical	Reduction of motor deficits, cognitive dysfunction and contusion volume.	Longhi et al., 2009 [59]

**sCR1**	C3 convertases	Classical, alternative, lectin	Reduction of neutrophil accumulation in the brain.	Kaczorowski et al., 1995 [68]

**Crry-Ig, GFAP-sCrry mice**	C3 convertases	Classical, altenative, lectin	Neuroprotection with improved neurological scores and decreased tissue injury and blood-brain barrier dysfunction.	Leinhase et al., 2006 [71] Rancan et al., 2003 [70]

**VCP**	C3b, C4b, C3 convertases	Classical, alternative, lectin	Improvement of sensorimotor outcome and spatial memory.	Pillay et al., 2007 [72] Hicks et al., 2002 [69]

**C5aR antagonist**	C5aR	C5a anaphylatoxin	Decreased neutrophil extravasation in the brain.	Sewell et al., 2004 [67]

See text for details and explanations.

Further therapeutic approaches were designed to more specifically target "key" effector components of complement activation, such as the anaphylatoxin C5a and its receptor (C5aR, CD88) [29,67,73,74]. In addition, more attention was recently devoted to target specific pathways of complement activation exclusively, in order to overcome the potentially deleterious effects of a complete "shut-down" of complement activation at the central C3 level. This notion is based on the fact that complement also mediates neuroprotective effects in the injured brain, as e.g. shown by a dose-dependent protection of glutamate-induced excitotoxicity against neurons by the C3-derived proteolytic fragment, anaphylatoxin C3a [75], and by C3a-mediated induction of nerve growth factor (NGF) by microglia [76]. Based on the recent concept of a "dual role" for complement in the pathophysiology of brain injury, by promoting both early neurotoxic and late neuroreparative mechanisms after TBI [12,77,78], the exclusive targeting of selected complement pathways was given more consideration, as opposed to the "pan" inhibition at the C3 convertase level [79-82]. Among these, the targeted inhibition of the alternative pathway has drawn particular attention in recent years [79,80,83]. Factor B, the "key" component of the alternative pathway, was previously reported to be significantly elevated in the intrathecal compartment of patients with severe TBI [65]. Experimental studies on factor B-deficient mice (*fB-/-*), which are devoid of a functional alternative pathway, revealed significant neuroprotection after closed head injury, in conjunction with a decreased extent of posttraumatic complement activation [79]. These positive findings derived from studies in gene knockout mice were extrapolated into a pharmacological approach, using a neutralizing monoclonal anti-factor B antibody (mAb1379) in the same model system [80]. The post-injury injection of mAb1379 led to significantly attenuated extent of complement activation and anaphylatoxin C5a generation, and was associated with an improved neurological recovery and reduced neuronal cell death after experimental closed head injury [80]. These data imply an important role of the alternative complement pathway in contributing to the delayed neuropathology after TBI, and provide strategic opportunities for therapeutic targeting of alternative pathway molecules as a potential future pharmacological strategy.

An additional avenue of research has been focusing on the terminal complement pathway, or "membrane attack" pathway, which results in cellular lysis by the MAC/C5b-9 [51,84,85]. In clinical studies, elevated levels of activated soluble MAC/C5b-9 were detected in the CSF of severely head-injured patients [62]. Moreover, the extent of intrathecal complement activation was associated with secondary cerebral insults in TBI patients, including post-injury BBB dysfunction [10,62,64]. Experimental studies have revealed that the intracerebroventricular injection of MAC induced a marked upregulation of adhesion molecule expression and leukocyte infiltration in the subarachnoid space and cerebral parenchyma [84]. In addition, MAC injection into hippocampus evoked seizures and neurocytoxic effects in rats [85]. Local MAC deposition in the injured brain was demonstrated in experimental models [86] and in injured human brains [87]. The complement regulatory molecule CD59 represents the main controlling molecule of MAC formation and an essential protector from neuronal cell injury after complement activation [51,88]. Neurons express CD59 constitutively, as a protective mechanism from autologous "innocent bystander" cell lysis after complement activation in the brain [51,89]. However, the posttraumatic activation of phosphatidyl-inositol-specific phospholipase C (PI-PLC) after traumatic brain injury renders neurons vulnerable to MAC-mediated lysis by shedding of the glycosyl-phosphatidyl-inositol (GPI)-anchored glycoprotein CD59 from neuronal membranes [88,90]. A recent experimental study on closed head injury in mice lacking the gene for *Cd59a *(CD59a^-/-^) revealed increased susceptibility to brain injury in *CD59a^-/- ^*mice, compared to wild-type littermates [88]. In fact, head-injured *CD59a^-/- ^*mice showed increased neuronal cell death in tissue sections assessed by TUNEL histochemistry, in conjunction with elevated serum levels of neuron specific enolase (NSE), an indirect marker of neuronal injury [88]. These data corroborate the crucial role of the complement regulatory molecule CD59 in protecting neurons from complement-mediated lysis, and emphasize the impact of the terminal complement pathway in contributing to the pathophysiology of delayed neuronal cell death after TBI.

Until present, there is a lack of specific pharmacological therapy designed to avoid induction of secondary brain injuries and delayed neuronal cell death [91]. There have been some significant advances in the field of therapeutic complement inhibitor development, in recent years [43,74,92-94]. While some of these inhibitors have been successfully tested in experimental head injury models (Table 1) [67,68,71,80], the "bench-to-bedside" extrapolation to clinical applications in head-injured patients has yet to be accomplished [91].

### Chest trauma and acute lung injury

Severe blunt chest trauma with associated pulmonary contusions is characterized by a robust inflammatory reaction which can result in exacerbated lung injury, acute respiratory distress syndrome (ARDS), multiple organ failure, and death [95-99]. Activation of alveolar macrophages and recruitment of neutrophils into the interstitial and alveolar compartments are followed by the release of an arsenal of proteinases and oxidants causing leakage of the pulmonary microvasculature and destruction of the alveolar epithelium [100-103]. Various experimental models of lung injury could yield important insights into the critical role of complement activation products, particularly anaphylatoxin C5a, in the pathophysiology of trauma-induced lung inflammation and progressive alveolar injury [28,104-106]. Elevated levels of C5a have been described in broncheoalveolar fluid samples from patients with acute lung injury [28,107,108]. When C5a was applied intratracheally in rats exposed to an IgG immune complex model, increased intrapulmonary generation of chemokines, accumulation of neutrophils and changes in vascular permeability could be detected [106]. The protective effects of anti-C5a were further corroborated by the observation that the antibody also suppressed release of tumor necrosis factor (TNF) into bronchoalveolar lavage [109]. Furthermore, C5a was shown to be required for TNF-dependent upregulation of intercellular adhesion molecule-1 (ICAM-1), an essential endothelial adhesion molecule required for neutrophil migration [109]. Czermak and colleagues demonstrated that both the *in vitro *and *in vivo *blockade of C5a led to significantly reduced production of CXC and CC chemokines [110,111].

**Figure 2:**
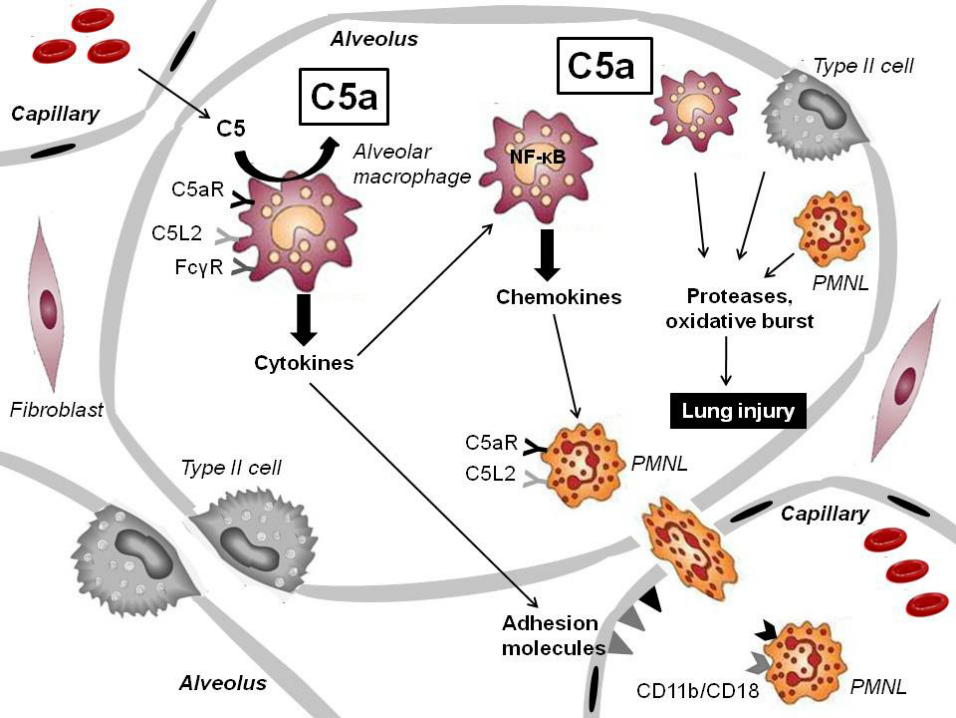
**Schematic understanding of complement anaphylatoxin C5a-mediated inflammation and alveolar injury after blunt chest trauma**. See text for details and explanations.

A proposed model for the current understanding of C5a-mediated inflammatory pathophysiology of acute lung injury is depicted in Figure 2. Anaphylatoxin C5a has been shown to induce the early release of pro-inflammatory cytokines by alveolar macrophages, such as TNF and interleukin (IL)-1β [104]. Interaction of endothelial adhesion molecules (e.g. ICAM-1) with their corresponding receptors on neutrophils (e.g. CD11b/CD18) leads to adhesion and transmigration of neutrophils into the alveoli [104]. Furthermore, release of TNF and IL-1β can also function in an autocrine way and activate alveolar macrophages to generate chemokines [112]. Among these, the different chemokines have been shown to further mediate neutrophil infiltration [113]. Activated neutrophils, alveolar macrophages and epithelial cells release reactive oxygen species and proteinases that cause diffuse alveolar and microvascular damage, thus exacerbating acute lung injury [111].

The interaction of C5a with its receptors, C5aR (CD88) and C5L2, is crucial for mediating the pulmonary inflammatory response. Bronchial and alveolar epithelial cells have been shown to express the C5aR [114,115]. Mice lacking the C5aR gene showed a decreased extent of pulmonary inflammation, as characterized by attenuated myeloperoxidase production by neutrophils and decreased vascular leakage [116].

Furthermore, the use of a specific C5aR antagonist led to similar attenuation of inflammation signs in immune complex-induced lung injury, indicating the C5aR as a predominant effector of the C5a-mediated inflammation in the lung [117]. A recent study could point out that the cellular responses induced by C5a/C5aR interaction are potentiated by a tight connection between complement and Fcγ receptors [118]. Both C5aR and FcγR are known to be expressed on alveolar macrophages [111]. Shushakova et al. found that C5a causes induction of the activating FcγRIII and suppression of the inhibitory FcγRII during lung injury resulting in a pro-inflammatory reaction. Genetic ablation of C5aR expression in mutant mice completely abolished C5a/C5aR-induced regulation of FcγRs and led to decreased intrapulmonary generation of TNF and neutrophil accumulation [118]. Taken together, C5a seems to have a broader critical function through FcγR regulation, thus augmenting inflammation in the lung. In contrast to the C5aR, the effects of C5a are limited by C5L2 that is co-expressed with the C5aR on many cells including neutrophils [119]. Besides of C5a, C5L2 can also bind C5a_desArg _and potentially additional complement fragments [120]. Gerard et al. could demonstrate a greater influx of inflammatory cells and an enhanced release of IL-6 and TNF in C5L2-deficient mice in the model of immune complex-induced lung injury [121]. This observation proposes an anti-inflammatory role of C5L2 in the lung that seems to counteract C5a/C5aR-mediated inflammation.

The complement-induced pulmonary response after chest trauma has been suggested to depend on a delicate balance between pro- and anti-inflammatory transcription factors [111]. Alveolar macrophage activation is characterized by increased nuclear translocation of nuclear factor(NF)-κB and activator protein-1 (AP-1) representing an initial event in the genesis of the inflammatory cascade [112,122]. In contrast to NF-κB and AP-1, the transcription factor STAT3 has emerged as a negative regulator of the inflammatory response [28]. Interestingly, C5a has been shown to be responsible for STAT3 activation in lungs and alveolar macrophages after immune complex-induced lung injury whereas no complement-dependence could be found for activation of AP-1 [122,123]. STAT3 has been hypothesized to act as a transcriptional mediator for the anti-inflammatory cytokine IL-10, and might contribute to a negative feedback system in acute lung injury [28,111,124]. In addition to the above described "classic" lung injury models, a recent study has paid more attention to the immune response after experimental blunt chest trauma induced by a blast wave [104]. Flierl and colleagues reported complement activation after trauma-induced bilateral lung contusion in rats with C5a-dependent perturbations in neutrophil functions. Treatment with anti-C5a antibody abolished functional deficits in neutrophils and reduced intrapulmonary levels of leukocytes and of cytokines [104].

Taken together, there is evidence from various animal models that support a predominant role of C5a in initiating a cascade of inflammatory events during acute lung injury. If lung trauma is severe, activation of the innate immune system can lead to a dysregulated inflammatory response resulting in ARDS [125]. Elevated levels of C3a and C5a were measured in plasma of patients with ARDS [126]. In addition, experimental complement inhibition led to attenuated pathology in an animal model of lung injury [126-128]. Thus, it is tempting to speculate that C5a might act as a potential target for immunomodulation after chest trauma [74], to avoid the deleterious effects of posttraumatic inflammation, which lead to ARDS, multiorgan failure, and death [97,129].

### Musculoskeletal trauma

Experimental models of musculoskeletal trauma demonstrated that the early posttraumatic inflammatory response is often accompanied by robust generation of complement activation products [66,104,105]. However, up to now, the involvement of the complement cascade in bone and cartilage trauma has only been marginally investigated [130]. In recent years, increased attention has been devoted to the investigation of the role of complement in bone biology and fracture healing [131]. Mesenchymal stem cells as progenitor cells of osteoblasts were shown to express the complement receptors C3aR and C5aR, and the complement regulator molecules, CD55 and CD59 [132-134]. Moreover, osteoblastic differentiation as a key aspect of bone formation and remodeling induces upregulation of a number of complement-related genes, like C1q, C4, C3aR, properdin, C1-inhibitor (C1-INH) and complement factor H [135]. Pobanz and colleagues reported the expression of a functional C5aR by a human osteoblast-like cell line and detected increased osteoblast IL-6 production after stimulation of these cells with C5a [136]. Furthermore, vitamin D3 has been described to regulate C3 production by murine osteoblastic cells both in vitro and in vivo [137-139]. Complement C3 was postulated to exhibit a modulating influence on the differentiation of bone marrow cells into osteoclasts [139,140]. Additional studies pointed out that complement appears to be involved in the transformation of chondral precursors to bone tissue during the enchondral ossification process, involving both the classical and alternative pathway complement activation [141,142]. Consequently, complement components were hypothesized to be also involved in the inflammatory response after musculoskeletal trauma, and in mediating induction of fracture repair processes [131]. A recent study revealed that the C5aR is expressed in fracture callus by differentiated osteoblast, chondroblast-like cells, and osteoclasts [143]. Since fracture healing is known to be delayed in case of additional trauma-induced injuries, it furthermore remains to be examined if systemic complement generation might be the initiator of this delayed recovery after musculoskeletal trauma [144].

In addition to the role in fracture healing, the effect of complement activation on cartilage destruction after joint injuries has been discussed in recent years [130]. Gene expression analyses demonstrated that chondrocytes express a broad range of complement components and complement regulatory proteins [145-147]. The origin of complement components in the synovial fluid remains a topic of debate [130,148]. Aside from chondrocyte-induced biosynthesis, it appears that multiple other non-cartilaginous sources contribute to complement release in the inflamed joint, including synovial cells and infiltrating leukocytes [130]. We recently hypothesized that chondrocytes may release pro-inflammatory cytokines, express neoantigens and undergo enhanced apoptosis after cartilage injury [130]. However, until present, the involvement of the complement system in posttraumatic joint inflammation and the development of posttraumatic osteoarthritis remains poorly understood, and requires further research.

**Figure 3:**
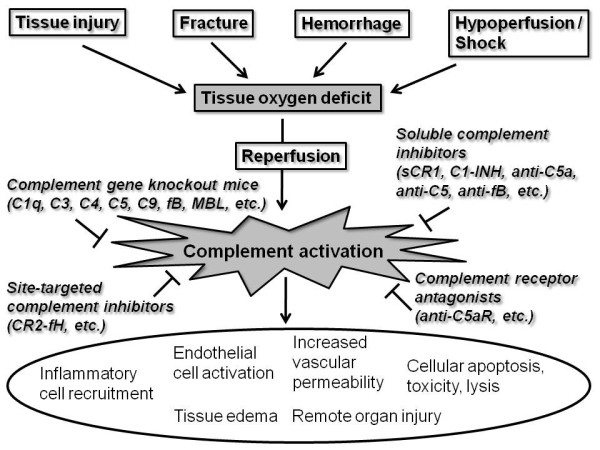
**Pathophysiology of complement mediated secondary tissue injury after major trauma, and potential pharmacological strategies for complement inhibition**. See text for details and explanations.

The pathophysiology of musculoskeletal trauma and of skeletal muscle ischemia/reperfusion is summarized in Figure 3. The oxygen deficit in major trauma, in conjunction with subsequent reperfusion of ischemic tissues has been recognized as a trigger of an intense inflammatory response that may cause damage both locally in the affected muscle and also in remote organs primary not involved in the ischemic insult [149-152]. Complement activation and consumption represents a critical event in the early phase of limb ischemia/reperfusion (I/R) injury resulting in the release of potent complement fragments like C3a and C5a [150,153,154]. It has been suggested that binding of preexisting natural IgM antibodies to neoantigen expressed by hypoxic cells after interruption of the blood flow is responsible for the activation of the classical complement pathway that importantly contributes to skeletal muscle I/R injury [155-157]. This hypothesis is strengthened by the fact that mice genetically deficient of mature B and T cells and natural antibodies (*Rag1^-/- ^*mice) show significant reductions of tissue damage in a model of hindlimb ischemia and reperfusion [155,158]. Furthermore, muscle edema and secondary neutrophil accumulation in the lung, as signs of reperfusion injury, were attenuated in *C1q^-/- ^*and *C4^-/- ^*mice deficient in central components of the classical complement pathway [159,160]. Aside from the classical pathway, recent data indicate important involvement of the classical and the lectin pathway in skeletal muscle I/R injury [159,161]. A protective effect was attributed to the complement regulatory molecules decay-accelerating factor (DAF/CD55), C1-INH, and soluble complement receptor type 1 (sCR1) after skeletal muscle reperfusion injury [162-164]. Moreover, a pivotal role of C5a in causing lung damage after hindlimb I/R was shown in an experimental study in rats [165]. In accordance with this observation, multiple markers of local and remote organ injury were markedly reduced in C5-deficient mice, and in mice treated with a neutralizing C5aR antagonist [74,166-168].

In summary, complement activation appears to play a significant role in contributing to post-injury inflammation in musculoskeletal trauma, including fractures, cartilage injury, and skeletal muscle I/R injury.

### Polytrauma and sepsis

Polytrauma is characterized as a syndrome of multiple injuries with defined severity which leads to a massive systemic immune activation and to secondary dysfunction and failure of remote, initially uninjured, organs [1,5-7]. Clinical studies have demonstrated that complement activation occurs in plasma of patients after major trauma, as early as at the time of presentation in the emergency department [169-171]. The extent of complement-mediated inflammation was correlated with injury severity, tissue hypoperfusion, and posttraumatic mortality [171,172]. Serum levels of C3 and C3a were identified as markers of injury severity and outcome in multiply injured patients [173,174]. Moreover, expression profiles of complement regulatory molecules and of the anaphylatoxin C5a receptor (C5aR/CD88) appeared to be significantly altered in leukocytes of multiply injured patients during the early phase of polytrauma, compared to blood samples from healthy volunteers [175]. The expression profiles of CD46 (membrane cofactor protein; MCP), CD59, and C5aR (CD88) on neutrophils correlated inversely with the severity of injury, an observation which was attributed to an intriguing trauma-induced "complementopathy" in multiply injured patients [175].

**Figure 4:**
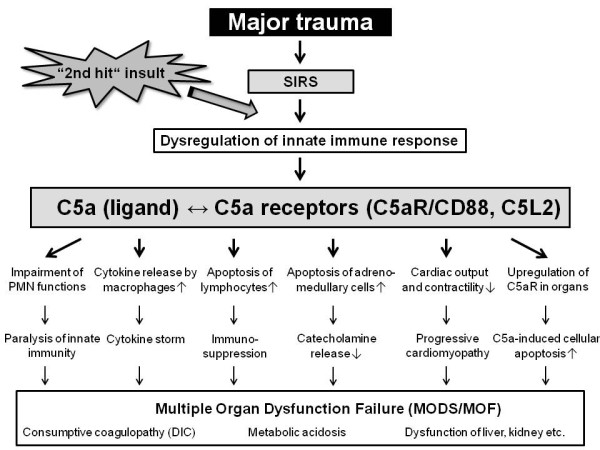
**Role of C5a ligand and receptor interaction in mediating the detrimental sequelae of major trauma, leading to secondary remote organ failure and adverse outcomes**. See text for details and explanations.

Sepsis represents a lethal complication of major trauma, characterized by an uncontrolled complement activation, as determined by significantly elevated plasma levels of C3a, C4a and C5a [176-178]. The anaphylatoxin C5a appears to represent the central molecule in the development of the overwhelming inflammatory response in sepsis, and has been coherently described as *"too much of a good thing" *[179-181] (Figure 4). Blockade of C5a was linked to improved survival in different experimental models of sepsis [182-185]. Persistent elevation of C5a during progressive sepsis was related to a posttraumatic immunparalysis with "shutdown" of crucial neutrophil functions, including a loss of chemotactic and phagocytotic activity, impairment of the oxidative burst, and disturbances in intracellular signaling pathways [48,186,187]. Recent studies corroborated an important contribution of C5a in modulating apoptosis in different cell types during sepsis. While apoptosis rates in neutrophils were shown to be significantly attenuated during sepsis, lymphocytes, thymocytes and adrenal medullary cells exhibited increased C5a-dependent susceptibility to programmed cell death [188-192]. The latter phenomenon was hypothesized to be responsible for impaired adreno-medullary catecholamine release predisposing the development of septic shock [191]. Excessive C5a levels during sepsis were furthermore associated with reduced myocardial contractility and cardiac output, a phenomenon described as "cardiomyopathy of sepsis" [193]. In general, multiple organs seem to be put at increased risk for C5a-mediated damage induced by an abrupt upregulation of the C5aR in a variety of tissues (heart, lung, kidney, liver, thymus) in early phases of sepsis [194,195]. A recent study implied that C5a-mediated signaling through the two C5a receptors (CD88 and C5L2) contributes to adverse outcome from sepsis [196,197]. In experimental models of sepsis, the blockade of C5a and its receptors has been shown to protect end-organ function and to improve outcomes, thus providing a future new avenue for pharmacological treatment of this detrimental complication of major trauma [198-201]. Future studies will have to be designed to validate this promising notion in a clinical setting.

## Conclusions

In recent years, multiple experimental and clinical studies have substantiated the notion of "key" role of complement activation after major trauma in contributing to the deleterious pathophysiological sequelae in the injured brain, lungs, and musculoskeletal system. Complement activation furthermore significantly contributes to the mechanisms of systemic post-injury complications, such as I/R injury, sepsis, and multiple organ failure. Therapeutic options aimed at attenuating the inflammatory complications of major trauma are currently unsatisfactory, and research strategies have largely failed in extrapolation from "bench to bedside". Experimental data from recent animal studies highlight the potential for complement inhibitors aimed at targeting central complement components and specific complement activation products, as promising future pharmacological agents in patients with major trauma. In this regard, site-targeted complement inhibition by new generation chimeric molecules which link pharmacological inhibitors to the local site of complement activation and tissue deposition may represent the future pharmacological "golden bullet". These chimeric molecules act locally at the site of injury and inflammation, and thus avoid the unwanted negative and adverse effects of a systemic complement blockade. Clearly, there is a tremendous need for well-designed experimental studies to shed some further light into our understanding of the complement-mediated pathology of major trauma, with the hope of designing and implementing new clinical treatment strategies for severely injured patients in the near future.

## Competing interests

The authors declare that they have no competing interests.

## Authors' contributions

PFS designed the concept of this article. MDN wrote the first draft. All authors contributed to revisions of the manuscript and approved the final version.
